# Supplementary material for: A Heme-Binding Transcription Factor BACH1 Regulates Lactate Catabolism Suggesting a Combined Therapy for Triple-Negative Breast Cancer
Source: Cells. 2022 Mar 31;11(7):1177. doi: 10.3390/cells11071177 (PMC8997507; doi:10.3390/cells11071177)
Supplement: Supplementary file 1 [file cells-11-01177-s001.zip › cells-1646000- supplementary.pdf]

Supplementary Figure S1.

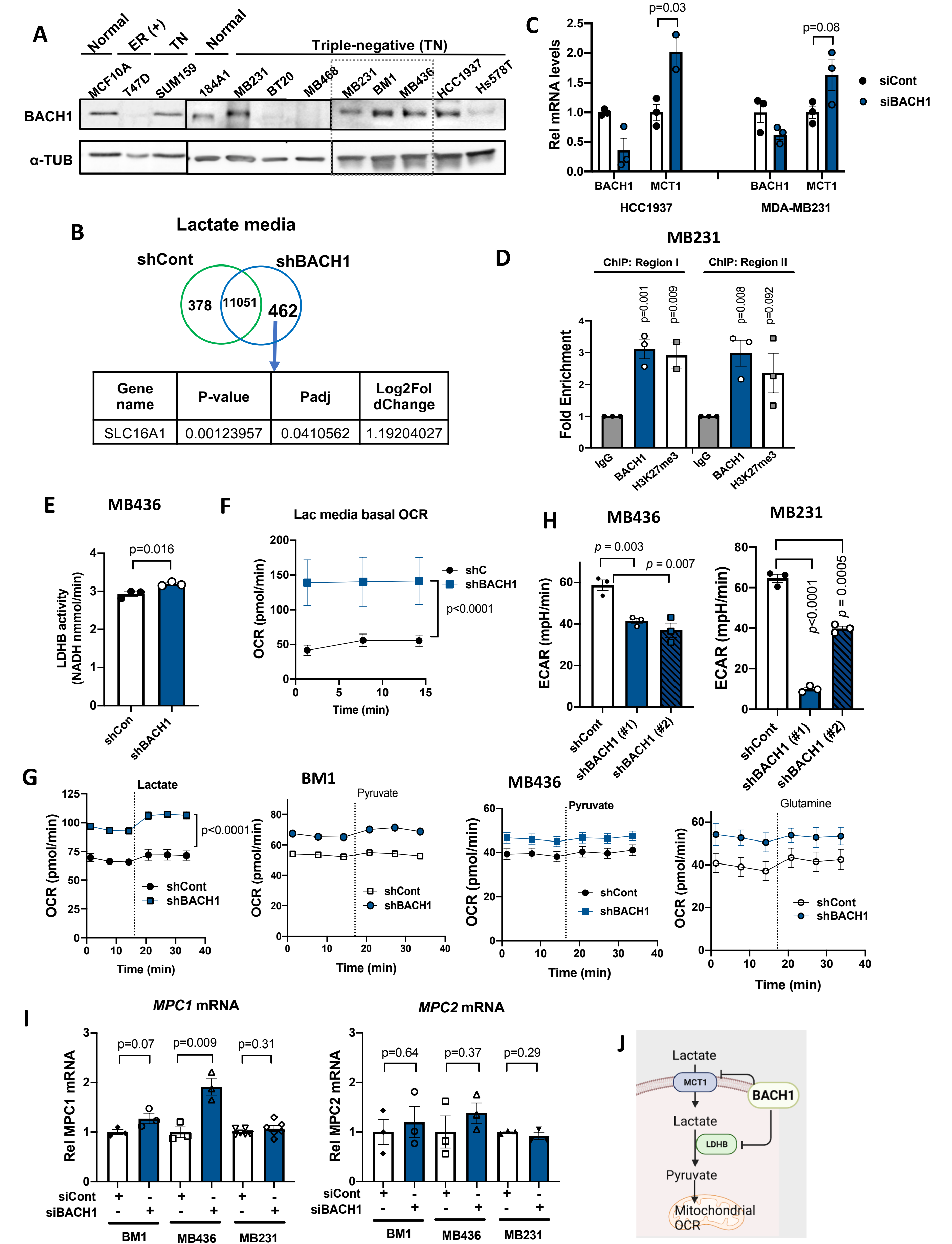

Supplementary Figure S1. BACH1 suppresses MCT1 transcripts and lactate-dependent mitochondrial metabolism in TNBC cells. (**A**) Representative protein blots showing relative BACH1 expression and alpha Tubulin as a loading control among TNBC cells (SUM159, MDA-MB-231, MDA-MB-231-BM1, BT20, MDA-MB-468, MDA-MB-436, HCC1937, Hs578T), one ER-positive T47D, and non-malignant breast epithelial MCF10A and 184A1 cells. (**B**) Venn diagram of differentially expressed genes in the transcriptomic analyses of control and BM1-shBACH1 cells (n=3/cell line) cultured in lactate (4 mM) supplemented growth media for 48 hours by RNA-sequencing. (**C**) Relative transcripts of *SLC16A1* mRNA of HCC1937 and MDA-MB-231 cells that are silenced for BACH1 using siRNA for 48 hours. (**D**) Relative fold changes of BACH1, H3K27me3, and IgG on the SLC16A1 promoter regions using MB231 cells. (**E**) Relative LDHB activity in control and MB436-shBACH1 cells. (**F**) Relative OCR of control and MB436-shBACH1 cells cultured in lactate-containing growth media. (**G**) Relative basal OCR of control and BM1-shBACH1 that are infused with either lactate (4 mM), pyruvate (2 mM), or glutamine (2 mM). (**H**) Basal ECAR measurement of control and MB436-shBACH1 or MB231-shBACH1 cells. Cells were cultured in the lactate-containing growth media and lactate was removed for ECAR measurement. (**I**) Relative mRNA of MPC1 and MPC2 in BM1, MB436, and MB231 cells transfected with siBACH1. (**J**) Schematic diagram showing lactate catabolic pathways suppressed by BACH1 in TNBC cells. For (**C**-**E**) and (**I**), Mean (n=3/cells) $\pm$ s.e.m., p-values by t-test. For (**F**) and (**G**), Mean (n=6/cells) $\pm$ s.e.m., p-values by t-test.

Supplementary Figure S2.

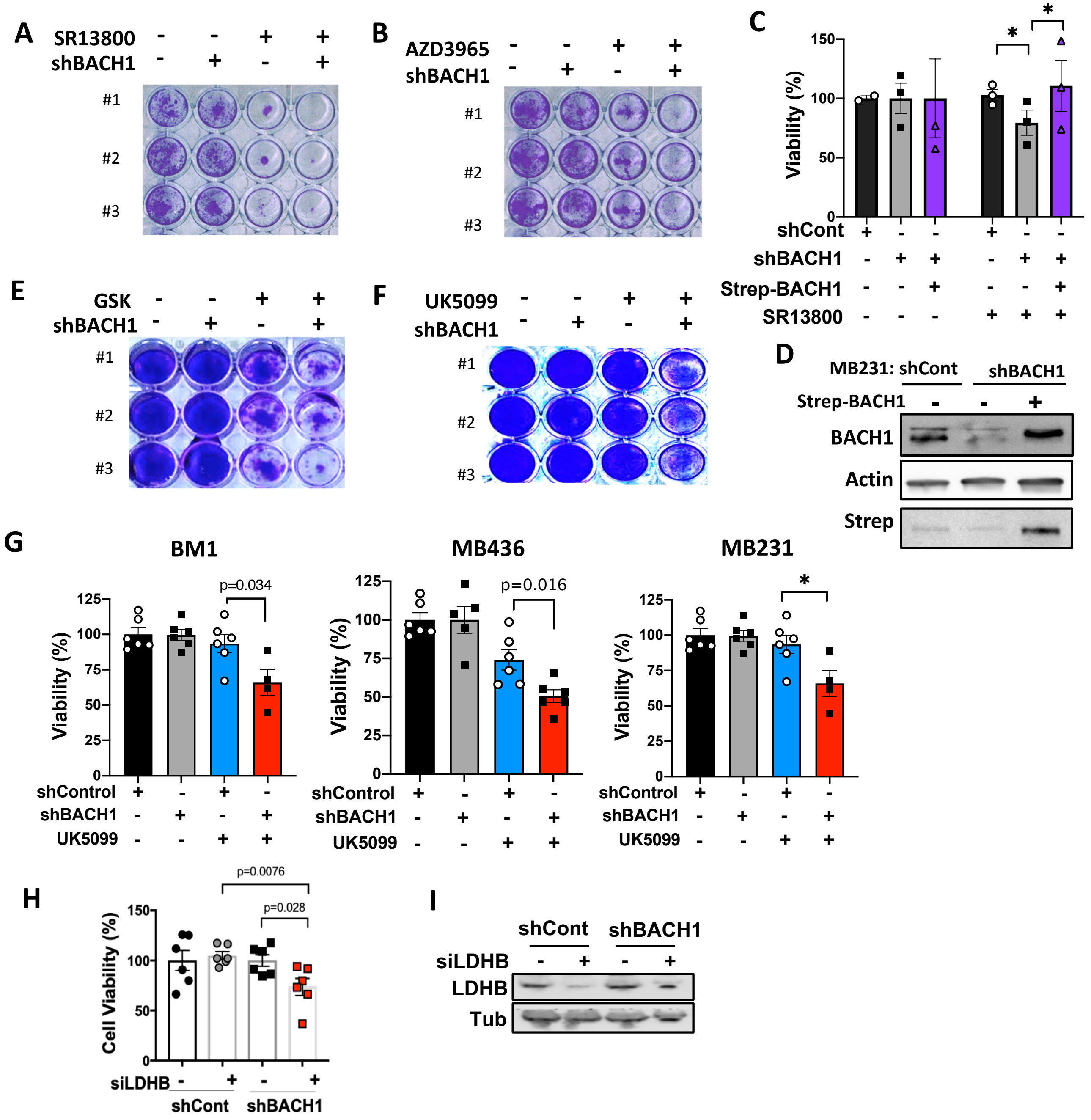

Supplementary Figure S2. BACH1 decreases efficacy of inhibitors targeting lactate metabolic pathways. (**A**, **B**) Colony formation of MB436-shBACH1 and control cells treated with SR13800 (50 μM) or AZD3965 (100 μM). After 48 hours of drug treatment, cells were further incubated in DMEM (25 mM glucose, 2 mM glutamine) media for 10 days, and stained using crystal violet. (**C**) Viability of MB231-shBACH1 and re-expressed BACH1 in shBACH1 cells relative to control cells that were treated with SR13800 (50 μM) for 48 hours. Mean (n=3/cells) $\pm$ s.e.m., p-values by t-test. (**D**) Representative protein blots of BACH1 of cell lines used for **C**. (**E**, **F**) Colony formation of BM1-shBACH1 and control cells were treated with GSK2837808A (200 μM) or UK5099 (100 μM) for 48 hours and cultured in high glucose DMEM. (**G**) Viability (%) of BM1-shBACH1, MB436-shBACH1, and MB231-shBACH1 cells that were treated with UK5099 (50 μM) relative to their controls (100%) using Calcein AM staining. Mean (n=3/cells) $\pm$ s.e.m., p-values by two-tailed t-test. (**H**) Viability (%) of BM1-shBACH1 cells that were silenced with LDHB using siLDHB (200 nM) or siScrambled (siCont) for 48 hours. Mean (n=6/cells) $\pm$ s.e.m., p-values by t-test. (**I**) Representative protein blots of LDHB and alpha-Tubulin of cell lines used for (**H**).

Supplementary Figure S3.

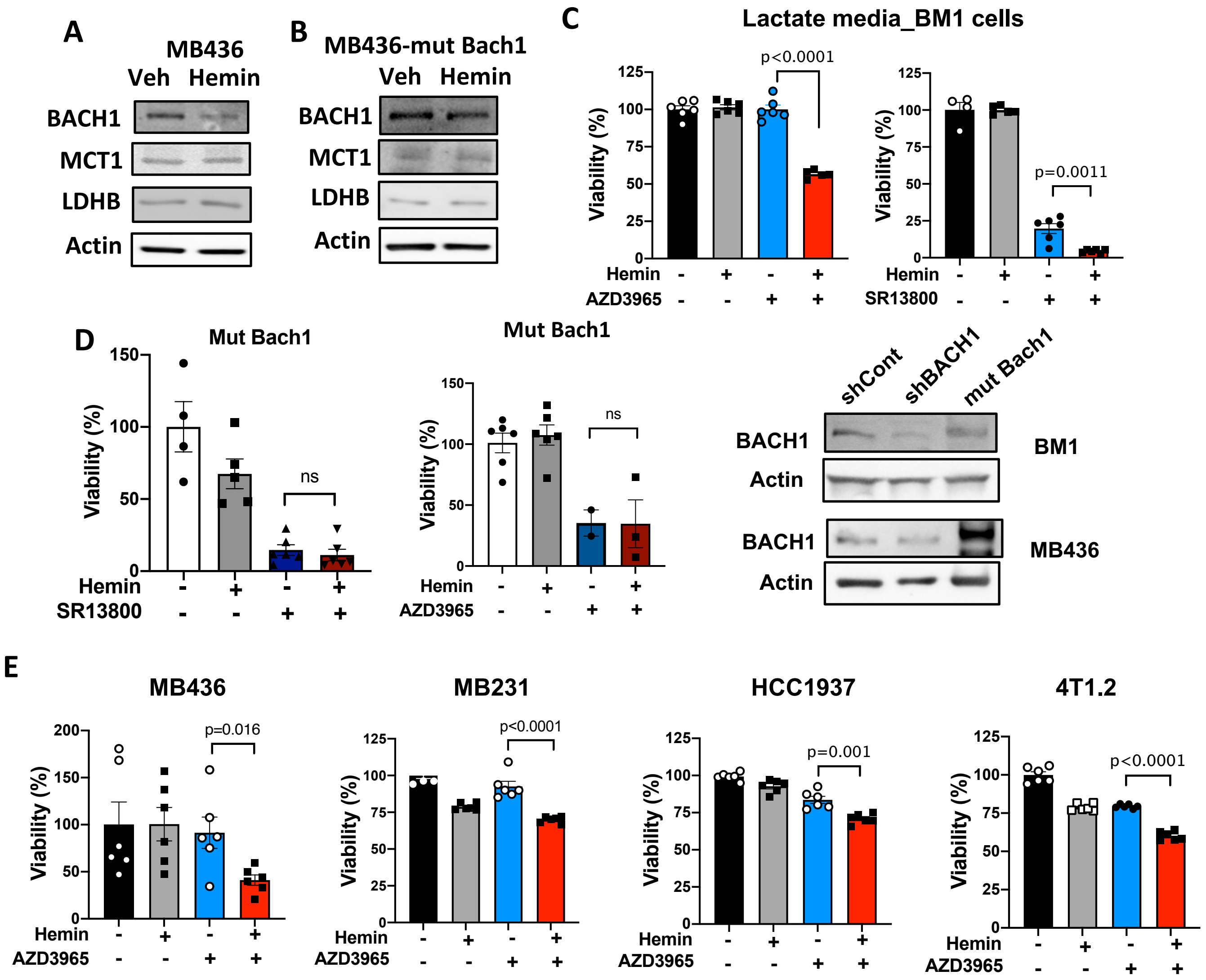

Supplementary Figure S3. Hemin increases efficacy of MCT1 inhibitors through BACH1 degradation in lactate sufficient environment. (**A, B**) Representative protein blots of BACH1, MCT1 and LDHB with a loading control beta-Actin using lysates of MB436 (**A**) or MB436-mut Bach1 cells (**B**) treated with hemin (20 μM) or vehicle for 48 hrs. (**C, D**) Viability (%) of BM1 (**C**) and BM1-mut Bach1 cells (**D**) that were treated with AZD3965 or SR13800 in combination with hemin (20 μM) or vehicle in lactate (4 mM) supplemented growth media (1.25 mM glucose DMEM) for 48 hrs and after Calcein AM staining (left). Protein blots showing relative BACH1 expression in the cell lines (right). (**E**) Viability (%) of MB436, MB231, HCC1937, and 4T1.2 cells that were treated with AZD3965 in combination with hemin (20 μM) or vehicle in lactate (4 mM) supplemented growth media (1.25 mM glucose DMEM) for 48 hrs and after Calcein AM staining. Mean (n=6/cells) $\pm$ s.e.m., p-values by two-tailed t-test.

Supplementary Figure S4.

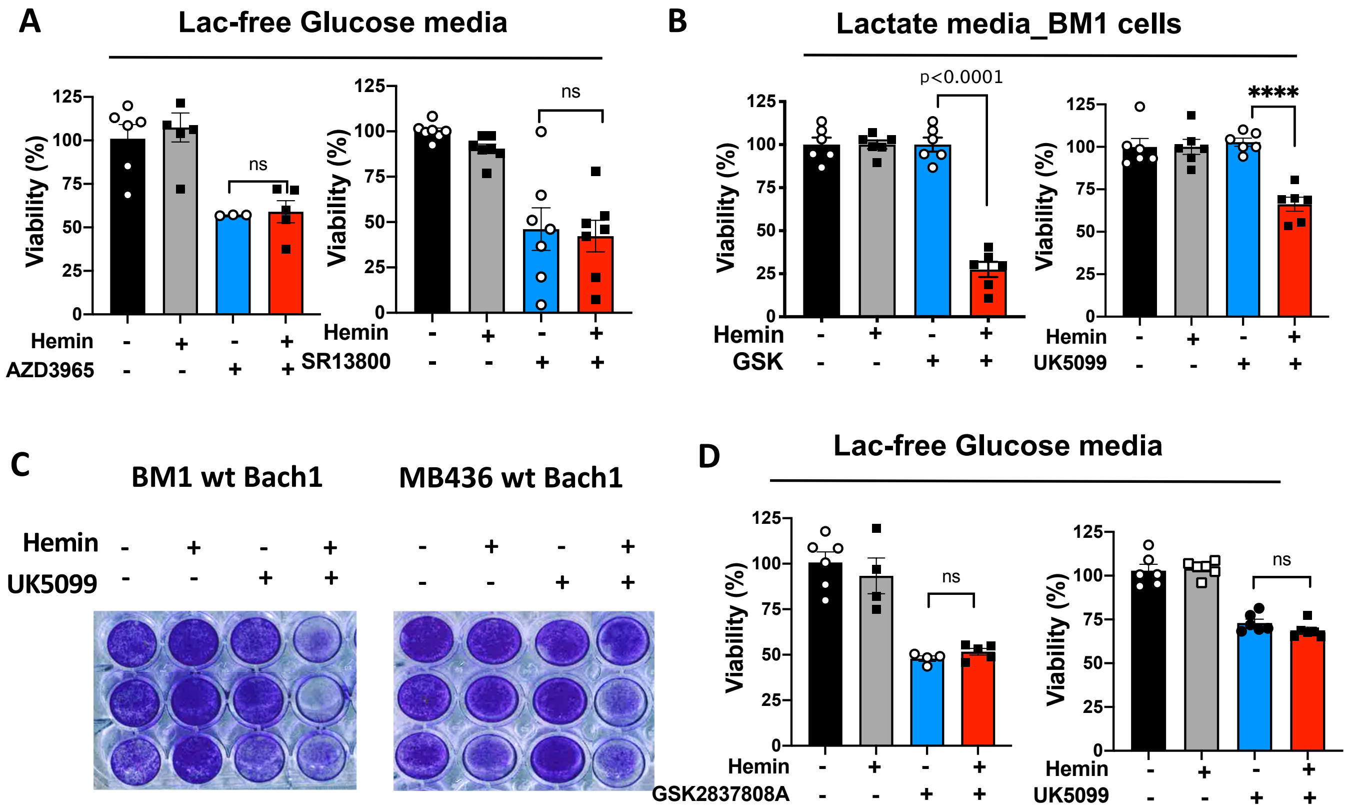

Supplementary Figure S4. Hemin increases sensitivity of cells to the inhibition of lactate catabolic pathways in lactate sufficient environment. (**A**) Viability (%) of BM1 cells that were treated with AZD3965 or SR13800 in combination with hemin (20 μM) or vehicle in lactate-free high glucose media (25 mM glucose, 2 mM glutamine DMEM) for 48 hrs and after Calcein AM staining. (**B**) Viability (%) of BM1 cells that were treated with GSK2837808A or UK5099 in combination with hemin (20 μM) or vehicle in lactate (4 mM)-supplemented growth media (1.25 mM glucose, 2 mM glutamine DMEM) for 48 hrs and after Calcein AM staining. (**C**) Representative colony formation of wt BACH1-expressing BM1 or MB436 cells that are treated with UK5099 (50 μM) and hemin (20 μM) for 72 hours and incubated in culture media (25 mM glucose, 2 mM glutamine DMEM) for 10 days. (**D**) Viability (%) of BM1 cells that were treated with GSK2837808A or UK5099 in combination with hemin (20 μM) or vehicle in lactate-free high glucose media (25 mM glucose, 2 mM glutamine DMEM) for 48 hrs and after Calcein AM staining. Mean (n=6/cells) $\pm$ s.e.m., p-values by two-tailed t-test.

Supplementary Figure S5.

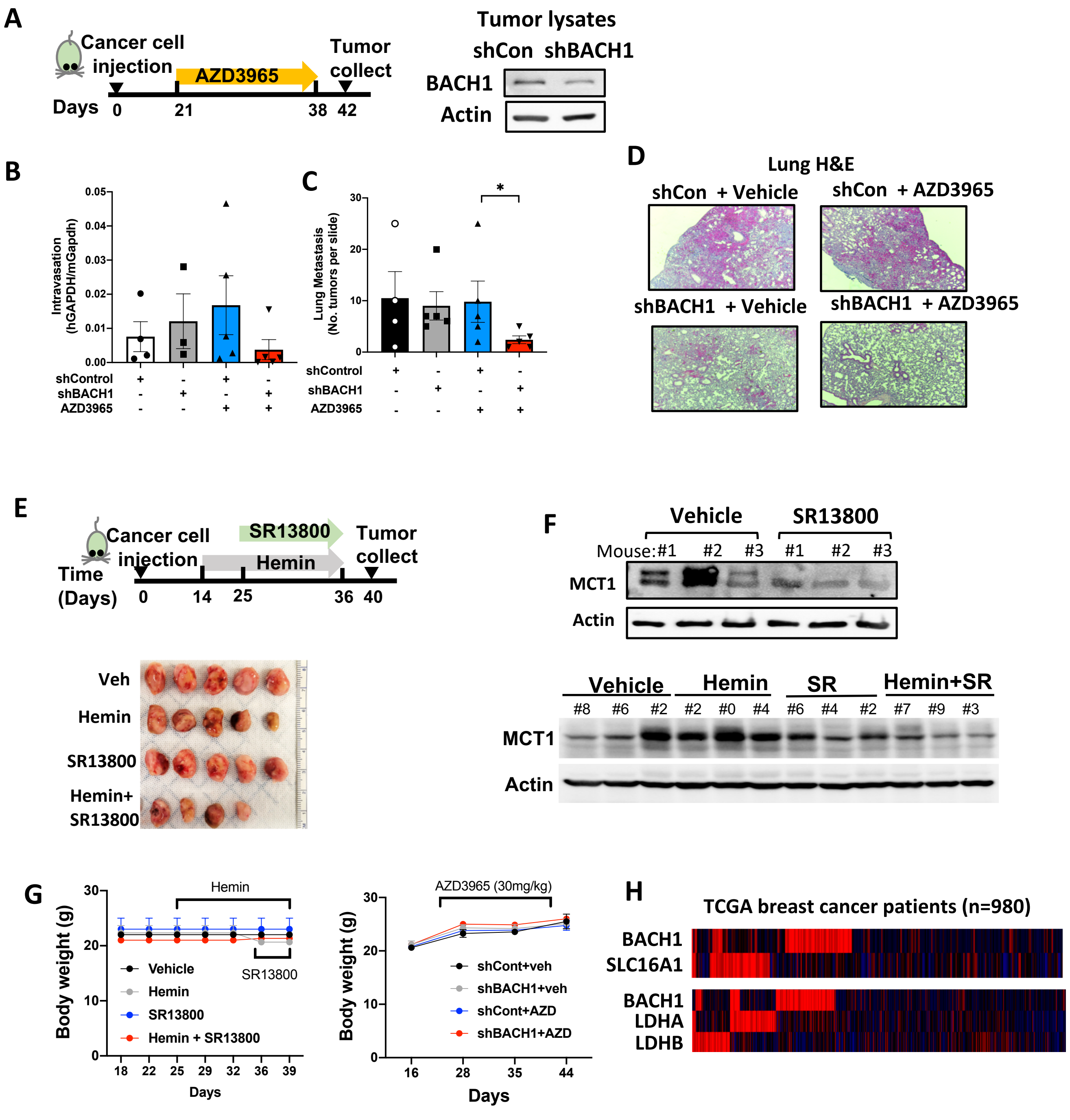

Supplementary Figure S5. Combining hemin with MCT1 inhibitors is effective to suppress breast cancer metastasis. (**A**) Diagram depicting mouse experiment (left) and protein blots (right) of BACH1 with a loading control beta-Actin using lysates from control and BM1-shBACH1 cells that were injected to athymic nude mice for xenograft models. (**B**) Intravasation measured by human GPADH mRNA relative to mouse Gapdh of serum isolated from xenografted mice using qRT-PCR (shControl $n = 4$, shBACH1 $n = 3$, shControl + AZD3965 $n = 5$, shBACH1+AZD3965 $n = 5$). (**C**) Lung metastases from mice with BM1-shBACH1 or control xenograft tumors. Lung tissues sectioned and H&E stained to visualize and count lung metastases in mice. (shControl $n = 4$, shBACH1 $n = 3$, shControl+AZD3965 $n = 5$, shBACH1+AZD3965 $n = 5$). (**D**) Representative lung metastasis images. (**E**) Diagram depicting hemin and SR13800 treatment for animal model (left) and representative tumor images (right) isolated from mice treated with either vehicle control, hemin, SR13800, or hemin+SR13800. (**F**) Representative protein blots showing MCT1 suppression after SR13800 treatment in mouse tumor lysates. (**G**) Body weight monitored before and after inhibitor treatment as indicated. (**H**) Heat map from OncoPrint analysis demonstrating expression (z-scores) of BACH1 and MCT1 (*SLC16A1*), or BACH1 and LDHA or LDHB for each patient with breast cancer (TCGA provisional dataset, $n = 980$).
